# Characterization of holins, the membrane proteins of coliphage ASEC2201: a genomewide *in silico* approach

**DOI:** 10.3389/fmicb.2025.1550594

**Published:** 2025-07-09

**Authors:** Humaira Saeed, Sudhaker Padmesh, Aditi Singh, Sujeet Pratap Singh, Mohammed Haris Siddiqui, Manodeep Sen, Imran Hussain, Mirza Masroor Ali Beg

**Affiliations:** ^1^Amity Institute of Biotechnology, Amity University Uttar Pradesh, Lucknow Campus, Lucknow, India; ^2^Dr. Ram Manohar Lohia Institute of Medical Sciences, Lucknow, India; ^3^Integral Institute of Agricultural Science and Technology, Integral University, Lucknow, India; ^4^Department of Bioengineering, Faculty of Engineering and Information Technology, Integral University, Lucknow, India; ^5^Faculty of Medicine, Alatoo International University, Bishkek, Kyrgyzstan

**Keywords:** transmembrane domain, phage-mediated lysis, ASEC2201, protein 3D structure, functional annotation, holins, bacteriophage, bacteriophage-encoded enzymes

## Abstract

Drug-resistant *Escherichia coli* poses a significant healthcare burden, driving the search for novel antimicrobials. We have previously done the isolation and whole-genome sequencing of ASEC2201, a novel coliphage derived from multidrug-resistant clinical *E. coli* strains. Here, we report the identification and characterization of phage enzyme, holin by in silico approaches. Genome annotation using Prokka identified three putative holin genes (PROKKA_03659, PROKKA_04292, and PROKKA_04422) belonging to the Phage_holin_2_1 superfamily. Upstream promoter prediction revealed active regulatory elements at positions 112, 177, and 186 for these genes, indicating robust transcriptional activity. Transmembrane topology analysis using DeepTMHMM confirmed the presence of two to three *α*-helical membrane-spanning domains in each holin, essential for pore formation. Homology modeling with SWISS-MODEL yielded high-confidence three-dimensional structures characterized by conserved membrane-anchoring motifs, as supported by QMEAN and GMQE quality scores. *In silico* identification of cell-penetrating peptide motifs within the holin sequences suggests potential for enhanced intracellular delivery in CPP-fusion therapeutic constructs. Overall, our in-depth analysis elucidates the structural and functional properties of ASEC2201 holins, underscoring their biotechnological significance as scaffolds for developing novel antimicrobial strategies against MDR *E. coli*. It gives us an understanding on how the holins, with their inherent membrane-disrupting functions, can be explored in detail for future use as lysis modules in programmable bacterial systems, while their identified CPP motifs offer additional potential for engineering targeted therapeutic delivery vehicles. This study also demonstrates the potential of integrative *in silico* approaches in developing a comprehensive foundation for future experimental validation for proteins with no prior functional annotation.

## Introduction

Holins and endolysins are crucial for host lysis in bacteriophage infections (Wang et al., 2000). Endolysins are muralytic enzymes that degrade the bacterial cell wall and accumulate in the cytosol, fully folded, during the vegetative cycle (Liu et al., 2023). Holins, small membrane proteins, accumulate in the bacterial membrane and, at a programmed time, create pores, allowing endolysins to access the cell wall (Abeysekera et al., 2022). This leads to the destruction of the bacterial murein and cell bursting. Holins regulate the timing of phage infection, which is under intense evolutionary pressure for optimal lysis timing. Their activity is controlled by protein inhibitors. Holins represent a highly diverse protein group, with over 100 known sequences forming more than 30 ortholog groups. Holins are classified into three classes based on their membrane topology. Class I holins, the largest, contain over 95 amino acids and form three transmembrane domains. Class II holins are smaller, with 65–95 amino acids, and form only two transmembrane domains. Class III holins are the smallest, with just one transmembrane domain. Class I and II holins are more commonly found than class III. These structural differences contribute to the varying mechanisms of pore formation and function within bacterial membranes, influencing the timing and efficiency of bacterial cell lysis during bacteriophage infection (Grabowski et al., 2021). However, on the basis of functionality, there are majorly three types of holins found, i.e.: canonical holins, pinholins and antiholins (Cahill and Young, 2019). In the context of bacteriophage infection, “canonical holins,” “pinholins,” and “antiholins” are all proteins involved in the cell lysis process, with the key difference being the size of the holes they create in the bacterial membrane. Canonical holins form large membrane pores, enabling endolysin release to degrade the cell wall, leading to cell lysis. Pinholins, in contrast, create smaller pinholes to depolarize the membrane, often acting before canonical holins initiate full-scale lysis.

Holins function as transmembrane transporters for endolysins, since endolysins cannot cross the bacterial membrane alone. After phage infection, precise timing and host lysis disrupt the cell envelope—inner membrane, peptidoglycan, and outer membrane. In the phage lytic system, holins form pores in the inner membrane to release endolysins and set the infection cycle’s endpoint. A threshold concentration of holins triggers pore formation, ensuring accurate lysis timing. By facilitating endolysin escape, holins accelerate and enhance host cell breakdown. Although phage-derived enzymes rarely face resistance due to extensive horizontal gene transfer, bacteria can resist exogenous endolysins via capsules, biofilms, and other outer defenses (Murray et al., 2021).

Antiholins regulate holin-mediated lysis by inhibiting pore formation until optimal timing is reached. In phage *λ*, dual translation of gene S yields holin S105 and antiholin S107; an extra N-terminal Lys residue hinders TMD1 membrane insertion, preventing early lysis, but membrane depolarization at trigger removes this barrier to activate lysis (White et al., 2010). In phage T4, cytoplasmic RIII binds the T holin’s N-terminal domain to delay pore formation, and upon superinfection, RIII activation establishes lysis inhibition, extending the latent period and boosting progeny maturation (Chen and Young, 2016; Krieger et al., 2020). Antiholins thus finely tune lysis timing, ensuring synchronized bursting with phage maturation.

Thus, such enzymes are necessary components of the lytic phage life cycle and provide a possible alternative to antibiotics (Singh et al., 2022). Endolysins have gained popularity in recent years because of their wide lytic activity against both Gram-positive and Gram-negative bacterial cells (Masih and Singh, 2023; Nandy et al., 2022). Using *in silico* technologies, researchers may examine the three-dimensional structural conformation of holins (Verma et al., 2024), categorize new domains (Yuan et al., 2023), investigate specific pathways to acquire a better knowledge of our evolutionary tree (Turner et al., 2021), identify more clusters (Zhang et al., 2023), and assign functions to the proteins (Zhang et al., 2025). This information may also be utilized to design effective pharmacological approaches and help in the creation of novel medications to treat a wide range of ailments (Setten et al., 2019; Ali et al., 2020; Kumar et al., 2021; Shaath et al., 2024). Lauer et al. (2010) developed genetically engineered *Listeria monocytogenes* strains expressing holin proteins to enhance the delivery of heterologous antigens into host cell cytosol. This strategy facilitates robust immune responses, making these strains promising vectors for vaccines and immunotherapies targeting cancer and infectious diseases. The approach is detailed in US Patent US20100129406A1, which describes pharmaceutical compositions and methods utilizing these modified Listeria strains (Lauer et al., 2010). Cell-penetrating peptides (CPPs) are promising tools for delivering therapeutic molecules, such as nucleic acids, drugs, and imaging agents, into cells and tissues in a nontoxic manner (Du et al., 2025; Pancholi et al., 2025). They are simple to synthesize, functionalize, and characterize (Copolovici et al., 2014), enabling delivery of bioactive cargos, including small drugs and large plasmid DNA, primarily via endocytosis (Moreno-Vargas and Prada-Gracia, 2024). CPPs facilitate processes like gene expression, silencing, and tumor targeting, making them valuable for future drug and diagnostic development (Borrelli et al., 2018; Zorko et al., 2022). However, their passive and nonselective nature often necessitates functionalization or chemical modification to achieve specific targeting (Nam et al., 2023). Designing effective systemic delivery systems requires careful consideration of both CPPs and their delivered cargos (Gautam et al., 2013; Ndeboko et al., 2020).

**Figure 1:**
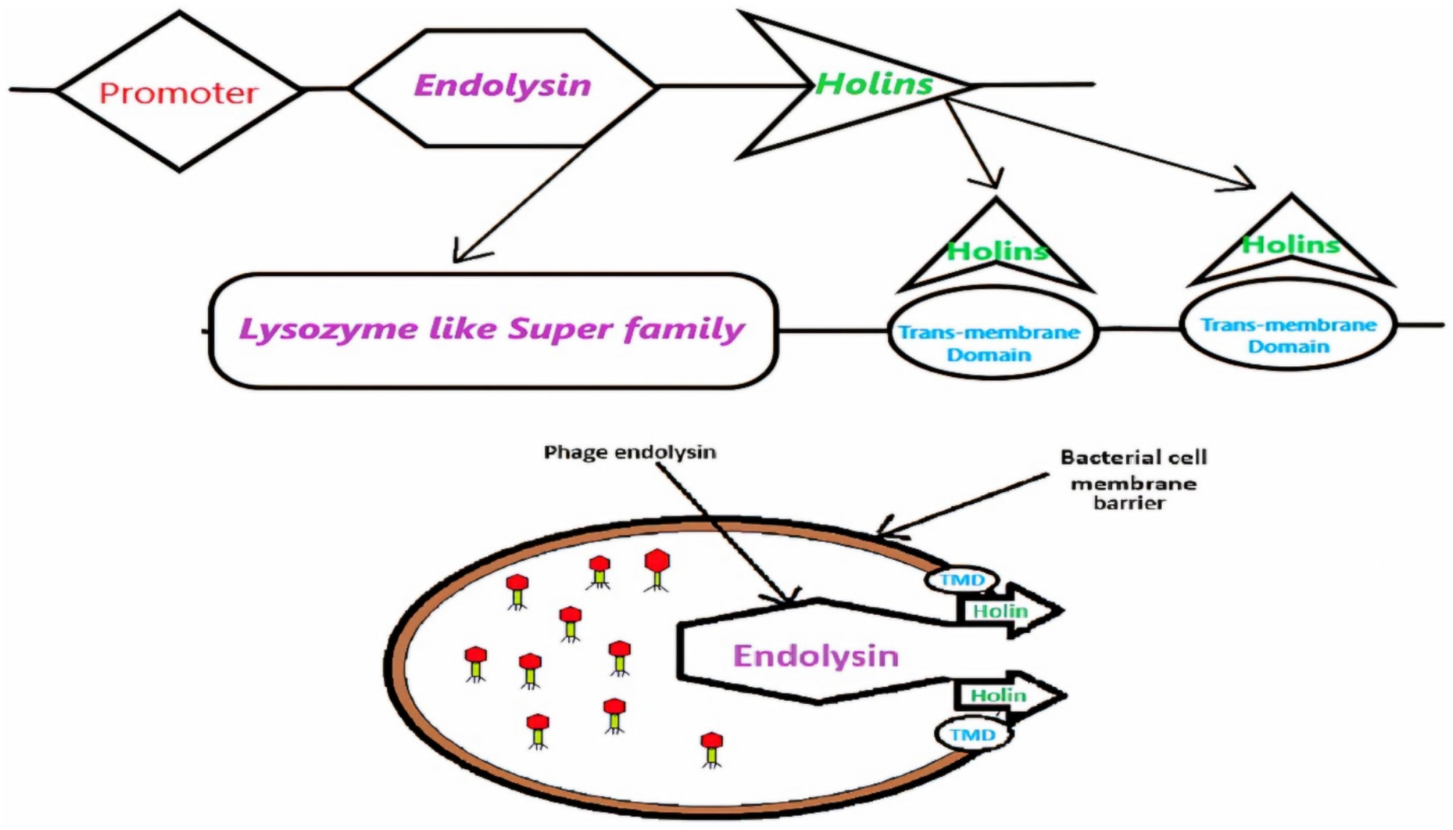
Transport of endolysin mediated by holin or the transmembrane domain (TMD), which results in different morphological changes in host cells.

In prior research, our laboratory identified two novel lytic bacteriophages, ASEC2201 and ASEC2202, from urban sewage, which effectively lysed 40–44% of *E. coli* clinical isolates and demonstrated resilience across diverse physicochemical conditions. Transmission electron microscopy classified them as tailed phages, and genomic analysis placed them within the Drexlerviridae family. In the current study, we conducted an *in silico* characterization of ASEC2201’s holins, focusing on their structural and functional attributes. This analysis provided insights into their transmembrane domains and supported their role in facilitating endolysin-mediated host cell lysis (Figure 1).

## Methodology

### Phage isolation, characterization, and genomic analysis

In our previous study, we have isolated and characterized a novel coliphage designated as ASEC2201. It has shown promising results for extreme environmental resilience and biocontrol efficacy (Padmesh et al., 2024). The phage retained infectivity after exposure to thermal cycles between −20°C and 62°C and across a broad proton concentration window (pH 4–10). Moreover, ASEC2201 particles exhibited 40–94% viability when challenged with organic solvents and sustained plaque-forming ability following treatment with anionic and cationic detergents. *In vitro* lytic assays demonstrated potent bactericidal activity against a laboratory reference *E. coli* strain and lysed 40–44% of multidrug-resistant clinical *E. coli* isolates.

After gradient purification, the phage was identified by Transmission electron microscopy, which characterized ASEC2201 as tailed phage. For genomic analysis, DNA from ASEC2201 was extracted following a modified protocol (Jakočiūnė and Moodley, 2018). High-quality DNA was used for Illumina NovaSeq 6,000 sequencing (RedCliff Labs, India). Libraries were prepared using PCR-based dual-indexing. Reads were quality-checked and assembled *de novo*; contigs were validated using BLAST. The whole-genome sequencing and comparative genomics assigned them to the genus of Escherichia phages within the family Drexlerviridae (order Caudovirales). Resulting data were deposited into the National Center for Biotechnology Information (NCBI) databases, with assembled genomes submitted to BioProject and raw FASTQ files to the Sequence Read Archive (SRA, 2004).

### Protein selection and sequence retrieval

Protein-coding gene annotation is typically a two-step process. Initially, Prodigal is employed to identify open reading frames (ORFs) by locating gene coordinates, but it does not infer gene function. To assign putative functions, Prokka performs hierarchical annotation by comparing candidate genes to curated protein databases. It begins with a user-supplied, high-confidence protein set, using BLAST+ for sequence similarity searches. If no match is found, it progresses to UniProt’s verified bacterial proteins, covering \~ 16,000 sequences, and then optionally to RefSeq proteins specific to the organism’s genus—capturing nomenclature consistency. When sequence-based annotation fails, Prokka applies profile-based searches using HMMER’s hmmscan to query against Pfam and TIGRFAMs databases. An e-value threshold of 10^−6^ is consistently applied to ensure significance. If no reliable match is found across all levels, the gene is designated as a “hypothetical protein.” This layered strategy maximizes annotation accuracy and functional insight across diverse bacterial genomes (Seemann, 2014).

The genome of coliphage ASEC2201 has been analyzed and three holin protein coding genes were selected. The sequences of all three holin proteins were retrieved from NCBI using accession no. SRX17770782 in the FASTA format. The sequence similarity search was performed via BLAST against the non-redundant database (Altschul et al., 1990).

### Phylogenetic analysis of ASEC2201 holin system

The annotated genome of coliphage ASEC2201 was retrieved from the NCBI database and analyzed for similarity using BLASTn. This allowed the identification of regions with homology and potential functional similarities. A phylogenetic analysis using the neighbor-joining method was conducted, Mega XII was employed to build a phylogenetic tree among three key proteins, providing insights into their evolutionary relationship (Kumar et al., 2018).

### Secondary structural properties and assessment

Secondary structure predictions were conducted using the following two step process. The amino acid sequences of the holin proteins were retrieved in FASTA format. SOPMA (Self-Optimized Prediction Method with Alignment) was used to predict the proportion of *α*-helices, *β*-strands, and random coils for each protein. The SOPMA web server was accessed at: https://npsa-prabi.ibcp.fr/cgi-bin/npsa_automat.pl?page=npsa_sopma.html and default parameters were used. In the next step, PSIPRED v4.0 (available at: http://bioinf.cs.ucl.ac.uk/psipred/), was used for secondary structure validation. Each protein sequence was submitted individually, and predictions were recorded based on the graphical output and raw data. Predicted percentages of helices, strands, and coils were compared across tools for consistency. No manual adjustments were made to the input data or output interpretation.

### Three-dimensional structure prediction and validation

Swiss Model and HHpred were utilized to predict the 3D (tertiary) structure of holins from phage ASEC2201 (Zimmermann et al., 2018; Söding et al., 2005) To validate the predicted structures, the Ramachandran plot analysis was conducted using the PROCHECK tool from the SAVES (v.6.0) program.

### *In silico* analysis of the selected proteins

The amino acid sequence composition, instability index, aliphatic index, and GRAVY index of the three holin proteins from phage ASEC2201 were analyzed using the ExPASyProtParam tool (Nedvedova et al., 2023; Wilkins et al., 1999). The theoretical isoelectric point (pI) was determined using SMS Suite (v.2.0). These properties help predict protein stability, hydrophobicity, and overall behavior under physiological conditions, aiding in understanding their structural and functional characteristics (Stothard, 2000).

### Functional annotation of the three holins

The conserved domains of the three holins from phage ASEC2201 were predicted using NCBI’s CD-search tool. Protein motifs were identified with ExPASy’s ScanProsite tool, revealing key functional patterns (de Castro et al., 2006) Additionally, the evolutionary relationships of these holins were analyzed using the SuperFamily program, which provides insights into the evolutionary history and relationships of the proteins by comparing them to known protein superfamilies (Wilson et al., 2009) These tools aid in understanding the structural and evolutionary aspects of these holins.

### *In silico* genome-wide analysis of lysis system

ClustalW2 was used to align the amino acid sequences of phage ASEC2201 with homologous proteins. The 3D structure of ASEC2201 was modeled using Phyre2, while conserved domains were identified via the NCBI conserved domain database (Kelley et al., 2015). Promoter regions were predicted using Bprom, helping to map functional and regulatory regions within the phage genome for further research and application (Solovyev and Salamov, 2011).

### Cell penetrating peptide identification

High-throughput techniques have advanced the discovery of cell-penetrating peptides (CPPs). CPP has been used to determine the penetrating peptides in the amino acid stretch of selected holins protein, combining *in silico* screening with experimental validation offers a cost-effective and practical approach, ensuring reliable, reproducible outcomes *in vitro*. CellPPD, a standalone tool for predicting and designing CPPs, has been utilized in this study to evaluate the cell-penetrating capabilities of selected peptides, streamlining the identification process. Such approaches significantly enhance CPP research, enabling the development of efficient peptide-based delivery systems for therapeutic and diagnostic applications (Gautam et al., 2013).

### Protein transmembrane domain recognition by deep transmembrane helices hidden Markov models (DeepTMHMM)

DeepTMHMM, a protein language model-based algorithm that can detect and predict the topology of both alpha helical and beta barrels proteins with unprecedented accuracy, has been used in the study. It scales to proteomes and covers all domains of life, which makes it ideal for metagenomic analyses (Hallgren et al., 2022). DeepTMHMM outperforms traditional HMM-based methods by integrating deep neural networks with biological sequence understanding and accurately predicts cytoplasmic vs. extracellular orientation of N- and C-termini. This is crucial for understanding holin activation, interaction with antiholins, and membrane-disruption mechanisms. The number and arrangement of predicted TMDs help classify holins into Class I, II, or III and is particularly effective in genome-wide mining for new holins or membrane proteins with limited sequence homology, using structure-based topology rather than relying solely on sequence alignment.

## Results

### Genome annotation of phage ASEC2201

The complete nucleic acid sequence of coliphage ASEC2201 was determined through next-generation sequencing (NGS) as described above. Subsequent *de novo* assembly of the raw sequencing data yielded the complete genome sequence. The genome was annotated with Prokka, which identified three holin protein-coding genes: PROKKA_03659, PROKKA_04292, and PROKKA_04422 (Supplementary Table 1).

### Phylogenetic analysis of holin proteins in coliphage ASEC2201

**Figure 2:**
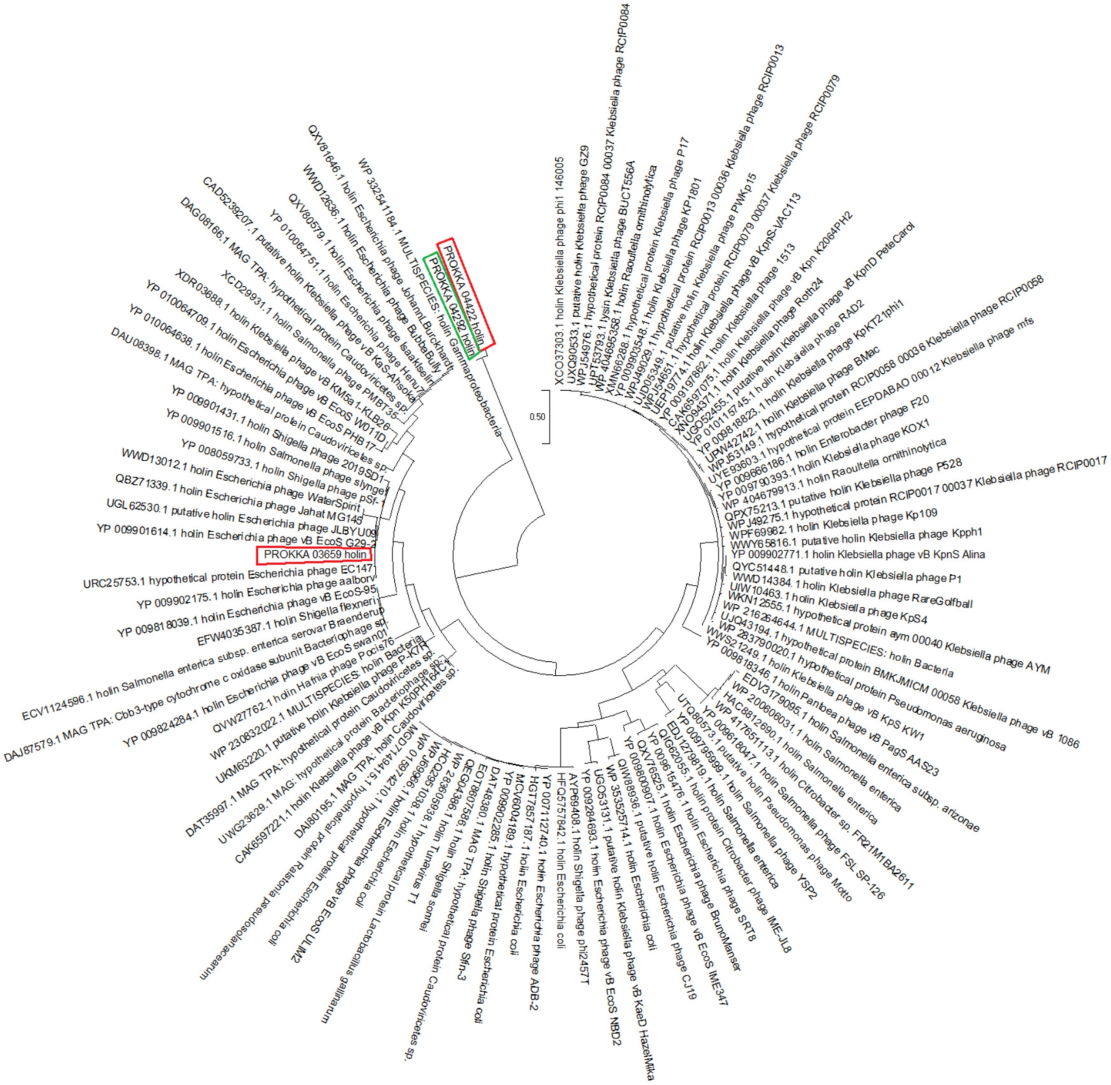
Phylogenetic analysis of PROKKA_03659, PROKKA_04292 and PROKKA_04422 by using neighbor joining method in MegaXII.

The un-annotated holins named PROKKA_03659, PROKKA_04292 and PROKKA_04422 fall into two quite different parts of the Gammaproteobacteria/phage-holin subtree (Figure 2).

PROKKA_03659 is nested deep within a well-supported subclade (bootstrap 1.00) of Escherichia phage holins. Its closest neighbors in that clade are experimentally characterized phage holins such as, YP_009901614.1 (holin from *E. coli* phage vB EcoS G29-2), UGL62530.1 (putative holin from *E. coli* phage JLBYU09), QBZ71339.1 (holin from *E. coli* phage Jahat MG145) and WWD13012.1 (holin from *E. coli* phage WaterSpirit). Clustering supports that PROKKA_03659 is very closely related to known Escherichia phage holins.

PROKKA_04292 and PROKKA_04422 actually form their own little sister-pair at the base of the Gammaproteobacteria holin radiation. The branch uniting them has only weak bootstrap support (~0.06), suggesting it is uncertain (i.e., it is a tentative placement). They sit immediately downstream of the broad “WP_332541184.1 holin Gammaproteobacteria” root, before the big cluster of Enterobacteria phage holins (including PROKKA_03659’s clade). In other words, PROKKA_04292 and PROKKA_04422 represent a distinct lineage of gammaproteobacterial holins that is not part of the main Escherichia phage grouping (Supplementary Figures 1, 2).

PROKKA_03659 is almost certainly a canonical Escherichia phage-type holin, given its tight clustering and high support with those sequences. PROKKA_04292 and PROKKA_04422, by contrast, look like they belong to a more divergent and as yet uncharacterized branch of Gammaproteobacteria-associated holins. Their basal position and low bootstrap suggest they may have quite different sequence/functional features compared to the well-studied enterobacterial phage holins.

### Secondary structure prediction by SOPMA

The secondary structure prediction of the holin proteins using SOPMA (Self-Optimized Prediction Method with Alignment) software provides valuable insights into their structural composition. Each output line contained 70 amino acids, and the sequence length was noted (Supplementary Tables 2, 3). Additionally, the amino acid composition was analyzed using the ProtParam Tool from ExPASy, providing insights into the properties of these holins, revealed that 55–63% of the secondary structures are in alpha helix forms. Alpha-helices are known for their stability and robustness to mutations, making them a common structural motif in proteins. The presence of *α*-helices is often associated with specific functional roles, such as membrane spanning and protein–protein interactions (Langosch et al., 2011). Amino acid composition analysis revealed the hydrophobicity, charge distribution, and potential post-translational modifications of these holins. All of which are crucial for understanding the protein’s behavior and interaction with other molecules.

### Determination and validation of tertiary-structure of the holin

**Figure 3:**
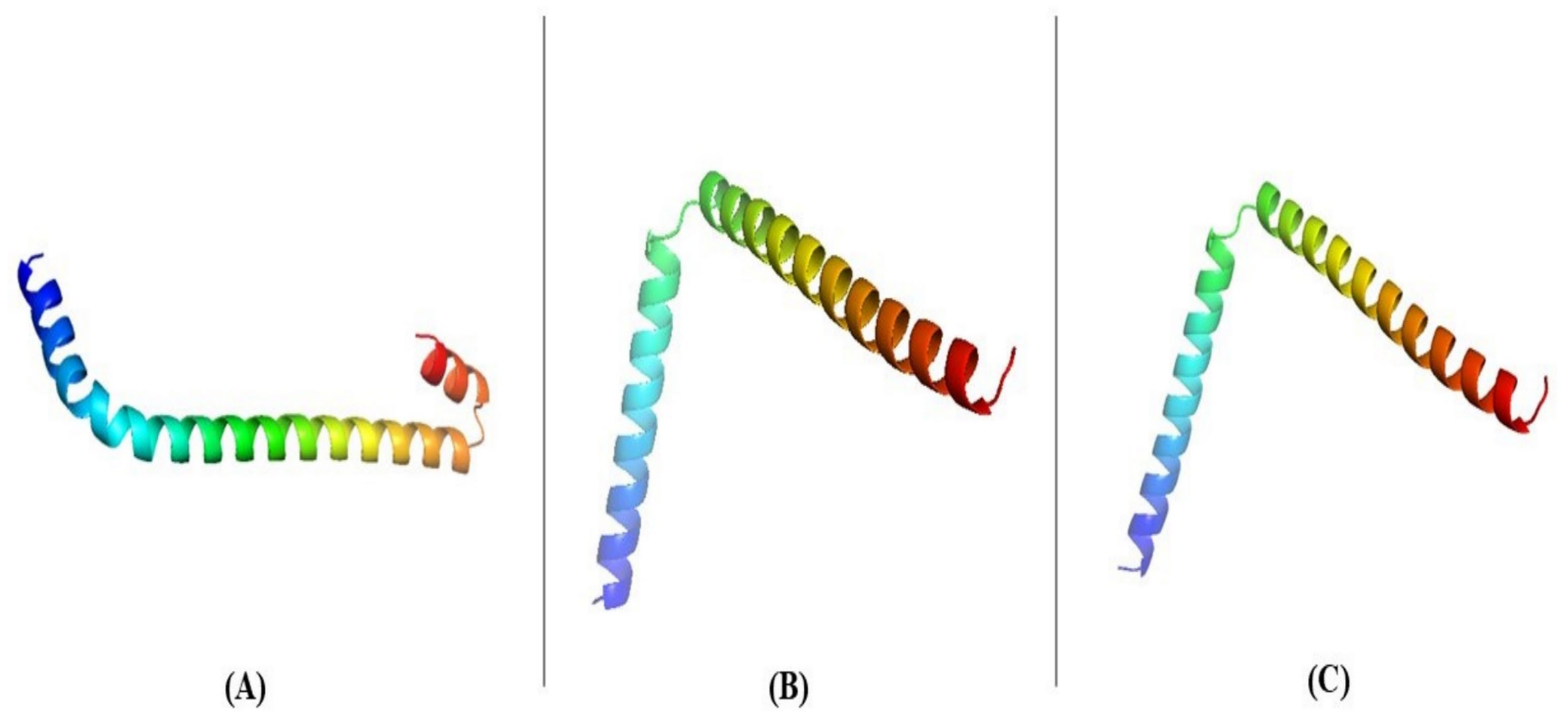
Tertiary structure of PROKKA_03659 **(A)**, PROKKA_04292 **(B)** and PROKKA_04422 **(C)** determined via the Swiss model and the HHpred tool.

**Figure 4:**
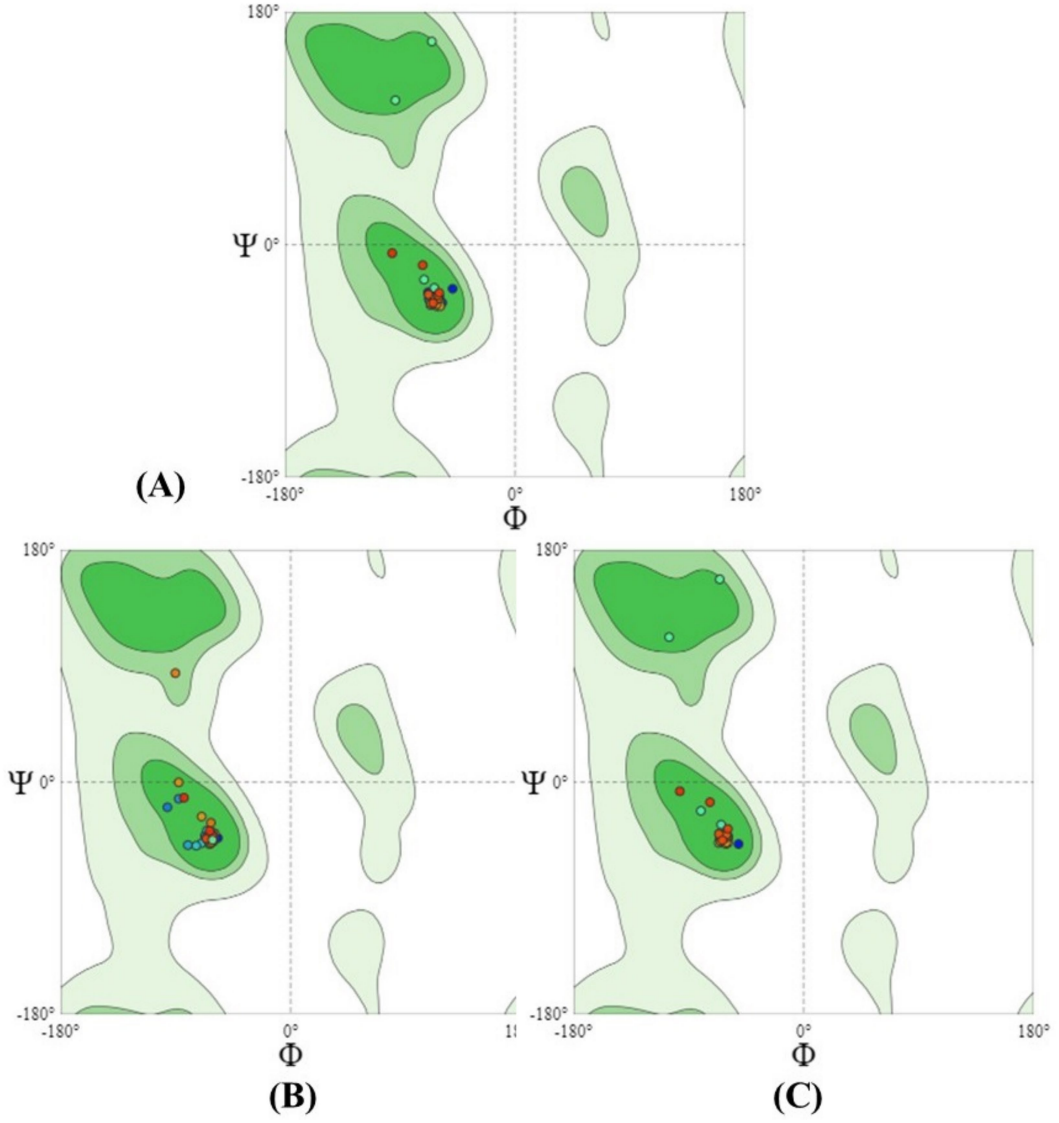
The PROCHECK program validated the Ramachandran plot statistics of the three-dimensional protein structure predicted by Swiss model. The graph’s red areas represent the locations that are most permissible. Green, light green, and white fields are, respectively, demarcated as additional allowed, generously allowed, and restricted regions [in the figure above, **(A)** is PROKKA_03659, **(B)** is PROKKA_04292 and **(C)** is PROKKA_04422].

The target sequence of the holin proteins in FASTA format was inserted into the HHpred Template Selection tool as the input, and the most suitable template (A0A655MZF6.1. A, P77237.1. A andP0A9R2.1. A) was selected with a probability rate of 100%, an E-Value of 2.4 × 10–116, a Cols of 342, and a target length of 372 and finally stored the tertiary modeled protein structure in PDB format, as predicted by Swiss model (Figure 3) (Gallego-Páramo et al., 2021). The Ramachandran plot by PROCHECK (Mondal et al., 2021) (Figure 4) was used to assess the holins’s tertiary structure, which revealed that for sequence PROKKA_03659; the 90 percent of the total residues (46) were found in the core (A, B, L); 7.5 percent of residues were in the additional allowed regions (a, b, l, p); and 0.0 percent of residues were in the generously allowed regions (a, b, l, p). The total number of non-glycine and non-proline residues were 40; that of the end-residues (excluding Gly and Pro) was 2; that of the glycine and proline residues was 4 and 0, respectively, out of 46 total residues. For the sequence PROKKA_0429296.9 percent of the total residues (38) were found in the core (A, B, L); no residues were found in the additional allowed regions (a, b, l, p); and in the generously allowed regions (a, b, l, p). The total number of non-glycine and non-proline residues was 32 and 2 respectively; that of the end-residues (excluding Gly and Pro) was 2; that of the glycine and proline residues was 3 and 1, respectively, out of 38 total residues. For the sequence PROKKA_04422 91.2 percent of the total residues (31) were found in the core (A, B, L); 8.8 percent residues were found in the additional allowed regions (a, b, l, p); and no residues were found in the generously allowed regions (a, b, l, p). The total number of non-glycine and non-proline residues were 3 and 1 respectively; that of the end-residues (excluding Gly and Pro) was 2; that of the glycine and proline residues was 3 and 1, respectively, out of 40 total residues (Supplementary Table 4). This analysis confirmed the quality and accuracy of the predicted structure by checking the distribution of dihedral angles in protein conformations, ensuring structural reliability (Laskowski et al., 1993).

The structural stability and favorable conformations of these holins imply that they are capable of adopting the necessary membrane-insertion conformations to facilitate pore formation in bacterial membranes (Lella et al., 2015).

### *In silico* analysis of the selected proteins

The physicochemical characterization of the three holins from coliphage ASEC2201 reveals essential insights into their stability and functional properties. The amino acid sequences were obtained in FASTA format and used for analysis. The stability indices of the holins indicated that all three proteins are stable, with PROKKA_03659 having an index of 28.95, PROKKA_04292 at 5.53, and PROKKA_04422 at 2.76, signifying their potential efficacy in therapeutic applications.

The theoretical isoelectric points (pI) for these proteins are in the basic range, with values of 9.43, 9.52, and 9.57, respectively, suggesting their roles may be influenced by pH conditions. Their aliphatic indices, above 66, indicate a high thermal stability, with PROKKA_03659, PROKKA_04292, and PROKKA_04422 values of 85.49, 97.65, and 92.11, respectively (Table 1).

**Table 1:** Physico-chemical parameters of holins of ASEC2201 characterized by *in silico* methods.

Parameters	PROKKA_03659 holin	>PROKKA_04292 holin	>PROKKA_04422 holin
No. of amino acids	71	68	71
Molecular weight	7482.68	7394.56	7778.06
Theoretical Pi	9.43	9.52	9.57
Total number of negatively charged residues (Asp + Glu)	6	5	5
Total number of positively charged residues (Arg + Lys)	9	8	9
Formula	C_331_H_534_N_92_O_97_S4	C_339_H_534_N_88_O_95_S_1_	C_358_H_563_N_89_O_100_S_2_
Total number of atoms	1,058	1,057	1,112
The estimated half-life	30 h (mammalian reticulocytes, in vitro). >20 h (yeast, *in vivo*). >10 h (*Escherichia coli*, *in vivo*).	30 h (mammalian reticulocytes, *in vitro*). >20 h (yeast, in vivo). >10 h (*Escherichia coli*, in vivo).	30 h (mammalian reticulocytes, in vitro). >20 h (yeast, in vivo). >10 h (*Escherichia coli*, in vivo).
Instability index (II)	28.95 (Stable)	5.53(stable)	2.76 (Stable)
Aliphatic index	85.49	97.65	92.11
Grand average of hydropathicity (GRAVY)	0.183	0.101	0.061

The GRAVY index values, all >0 (0.183, 0.101, and 0.61), indicate these proteins are hydrophobic, suggesting a likelihood of less water interaction and potential membrane association. These characteristics highlight the promising nature of these holins as candidates for further exploration in phage therapy and biocontrol applications (Singh et al., 2022).

### Protein homology/analogy recognition

**Figure 5:**
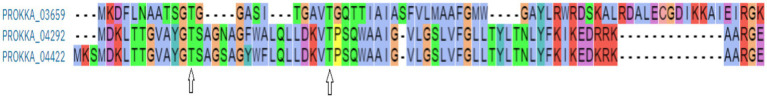
Catalytic triad sequence similarity in PROKKA_03659, 04292, and 04422 (shown here with an arrow sign).

The structural analysis of the coliphage holin proteins, predicted using the Protein Homology/Analogy Recognition Engine - Phyre2, reveals significant insights into their functional characteristics (Supplementary Figure 3). Phyre2 modeling suggests that all three holins adopt predominantly helical conformations, indicative of *α*-helical transmembrane domains. This structural feature is characteristic of holin proteins, which typically function by forming pores in bacterial membranes, facilitating the release of endolysins and subsequent cell lysis. Further comparative analysis indicates that these holins exhibit high structural homology to known proteins: PROKKA_03659 aligns with c8vxqB_, PROKKA_04292 with c8jjrm, and PROKKA_04422 with c6hwhX. These homologous relationships suggest that the ASEC2201 holins share evolutionary lineage with established holin families, providing a framework for understanding their functional roles and potential applications in bacteriophage therapy. The identification of conserved residues, such as Glu-2, Asp-4, and Thr-6, forming a (Figure 5 and Table 2) analogous to that found in lysozymes, further underscores the functional significance of these proteins. While holins are not enzymes themselves, the presence of such conserved motifs may indicate a regulatory mechanism or interaction with enzymatic partners, enhancing the efficiency of the lytic process.

**Table 2:** Catalytic triad of ASEC2201 holins.

Key residues	PROKKA_03659	PROKKA_04292	PROKKA_04422
Glu (E)	2	2	2
Asp (D)	4	3	3
Thr (T)	6	6	6

### Functional annotation of the selected holins

The identification of a conserved domain in the three holin proteins from coliphage ASEC2201 underscores their functional significance within the phage lifecycle. Utilizing the NCBI Conserved Domain Database (CDD) and CD-Search, which employs RPS-BLAST to compare sequences against position-specific scoring matrices derived from conserved domain alignments, a conserved domain was detected in these holins corresponding to the Phage_holin_2_1 superfamily (Pfam accession no. pfam04971). It has two transmembrane segments with both the N- and C-termini on the cytoplasmic side of the inner membrane in *E. coli*. Members of this family fall into the holin superfamily II, and Phage 21 S holin is the prototype for this superfamily. Class II holin family protein which is similar to *E. coli* prophage lysis protein S homolog EssQ and prophage lysis protein S homolog EssD, meaning *E. coli,* the prophage-encoded proteins EssQ and EssD are homologous to Class II holins. EssQ is a predicted membrane protein, while EssD is a hypothetical protein associated with the DLP12 prophage (Srividhya and Krishnaswamy, 2012). These proteins are believed to function in the lysis process during prophage induction. The EssQ protein is predicted to have a role in the lysis of the host cell, similar to other holin-like proteins (Wang et al., 2023).

### *In silico* genome-wide analysis of lysis system

The study of holins of coliphage ASEC2201 involved several bioinformatic tools to analyze its protein and genetic structure. ClustalW2 was employed to align its amino acid sequences with homologous proteins, providing insight into evolutionary relationships. For membrane topology and signal peptide predictions, TMHMM and the SignalP server were used, identifying potential transmembrane domains (Petersen et al., 2011). The 3D structure of holins of ASEC2201 has been predicted using Phyre2, a protein modeling tool. Conserved domains were detected via the NCBI Conserved Domain Database (CDD), which helped pinpoint functional elements within the proteins. Promoter regions were predicted using Bprom, aiding in understanding regulatory sequences (Table 3). Such a multi-layered analysis helps unravel phage biology and potential therapeutic applications (Wu et al., 2021).

**Table 3:** Promoter predictions of the modeled holin proteins using BPROM.

Promoter parameters	PROKKA_03659	PROKKA_04292	PROKKA_04422
Sequence length	216	207	216
Number of predicted promoters	1	1	1
Promoter position	112, LDF 0.74 No such sites for promoter at 112	177, LDF 2.18 rpoD17: AATCTTTA at position 154 Score- 7 purR: ATTTCAAG at position 161 Score- 9	186, LDF 2.40 purR: ATTTCAAG at position 170 Score- 9 soxS: GATAAGCG at position 187 Score- 9

### Cell penetrating peptide predictions

Cell penetrating peptides have been identified and selected using CPP web server. Six peptide stretches were identified in PROKKA_03659, PROKKA_ 04292 and four peptide stretches have been identified in PROKKA_04422 holin protein, respectively (Table 4). These peptides, located predominantly in the N-terminal bactericidal regions of the proteins, exhibit characteristics typical of CPPs, such as a high density of positively charged amino acids. This structural feature facilitates their ability to traverse cellular membranes efficiently.

**Table 4:** Cell penetrating peptide predictions of selected holin proteins using CPP web server and their peptide stretches.

Holins’ sequence	Peptide Sequence	SVM score	Prediction	Hydrophobicity	Hydropathicity	Hydrophilicity	Charge	Mol wt
PROKKA_03659	GMWGAYLRWR	0.03	CPP	−0.14	−0.54	−0.67	2.00	1295.67
	MWGAYLRWRD	0.03	CPP	−0.23	−0.85	−0.37	1.00	1353.70
	GAYLRWRDSK	0.26	CPP	−0.43	−1.42	0.43	2.00	1251.54
	YLRWRDSKAL	0.07	CPP	−0.39	−1.00	0.25	2.00	1307.65
	LRWRDSKALR	0.24	CPP	−0.57	−1.32	0.78	3.00	1300.66
	RWRDSKALRD	0.09	CPP	−0.69	−2.05	1.26	2.00	1302.58
>PROKKA_04292 holin	GFWALQLLDK	0.07	CPP	0.05	0.38	−0.56	0.00	1190.56
	YFKIKEDRRK	0.05	CPP	−0.68	−2.17	1.44	3.00	1382.76
	FKIKEDRRKA	0.21	CPP	−0.66	−1.86	1.62	3.00	1290.66
	KIKEDRRKAA	0.32	CPP	−0.69	−1.96	1.82	3.00	1214.56
	IKEDRRKAAR	0.09	CPP	−0.76	−2.02	1.82	3.00	1242.57
	KEDRRKAARG	0.07	CPP	−0.82	−2.51	2.00	3.00	1186.46
>PROKKA_04422 holin	YFKIKEDKRK	0.07	CPP	−0.61	−2.11	1.44	3.00	1354.75
	FKIKEDKRKA	0.22	CPP	−0.59	−1.80	1.62	3.00	1262.65
	KIKEDKRKAA	0.25	CPP	−0.63	−1.90	1.82	3.00	1186.55
	KEDKRKAARG	0.02	CPP	−0.75	−2.45	2.00	3.00	1158.45

The presence of CPPs in holin proteins suggests their potential as vectors for intracellular delivery of therapeutic agents. Due to their positive charge, these peptides can form complexes with negatively charged molecules like nucleic acids, enhancing the delivery of genetic materials into cells. Additionally, CPPs can be conjugated with various therapeutic biomolecules, including peptides, proteins, and antimicrobial drugs, thereby expanding their utility in targeted drug delivery systems.

### Protein transmembrane domain recognition by deep transmembrane helices hidden Markov models (DeepTMHMM)

**Figure 6:**
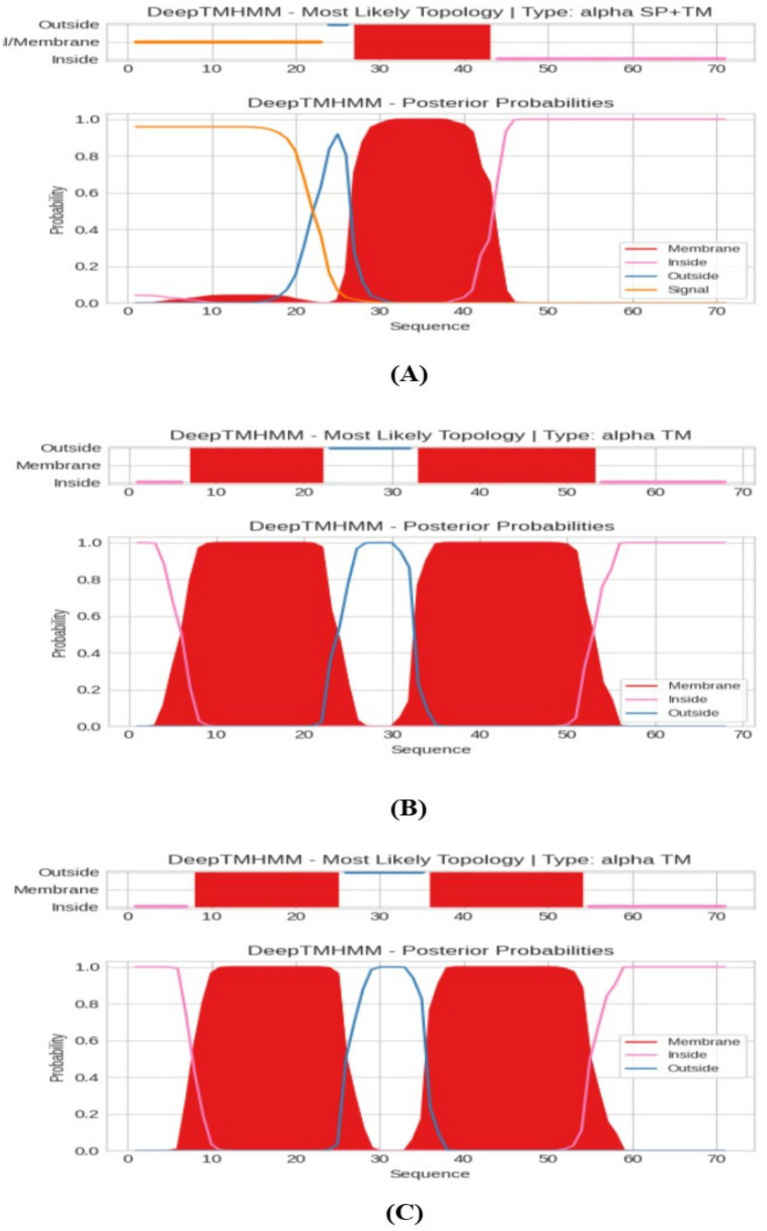
Protein transmembrane domain recognition by deep transmembrane helices hidden Markov models (DeepTMHMM), where **(A)** represents the result of PROKKA_03659, **(B)** for PROKKA_04292 and **(C)** for PROKKA_04422, respectively.

**Figure 7:**
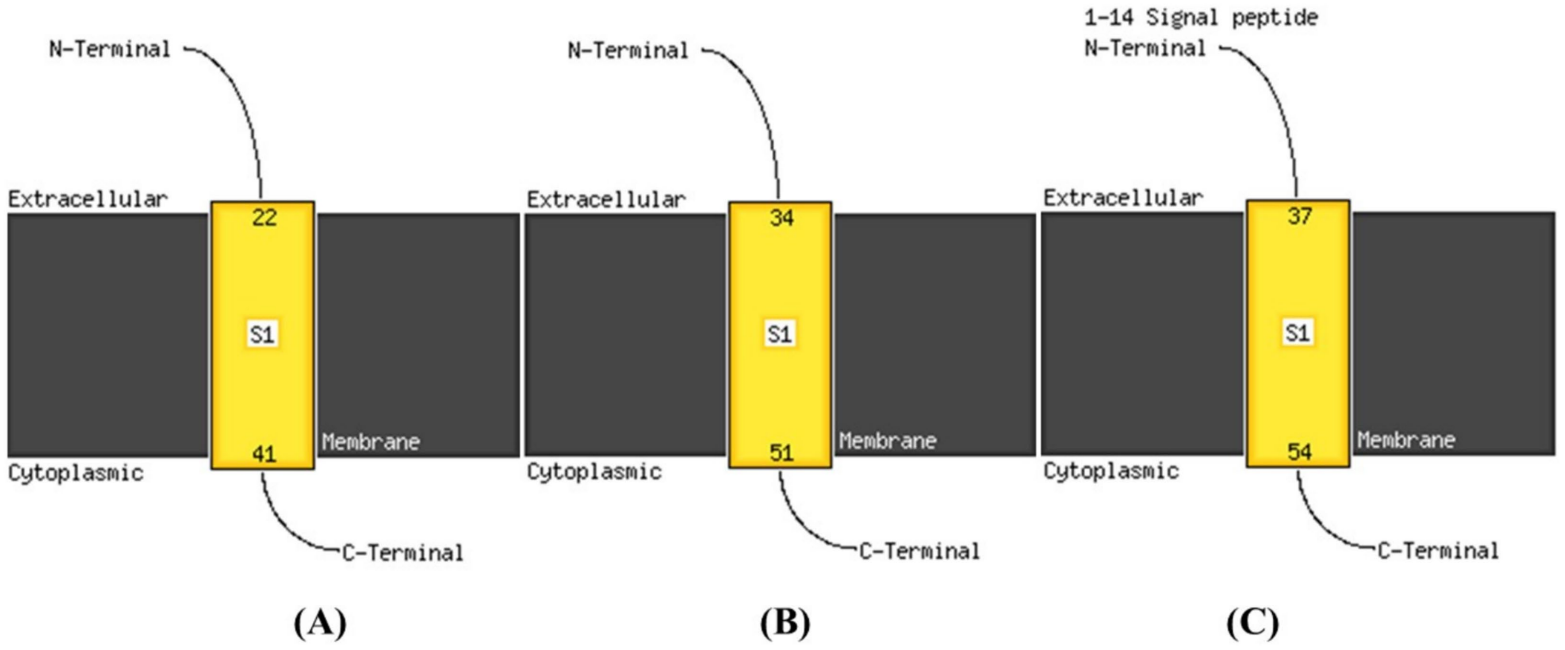
Predicted transmembrane N-terminal regions of annotated proteins PROKKA_03659 **(A)**; PROKKA_04292 **(B)**; PROKKA_04422 **(C)**, via Phyre2 Modeling.

Transmembrane proteins span the lipid bilayer and are divided into two major structural classes, namely alpha helical and beta barrels. TMHMM analysis as represented in Figure 6 indicated that PROKKA_03659 contains a single transmembrane domain (TMD), while PROKKA_04292 and PROKKA_04422 each possess two putative TMDs. Holins are classified into three groups based on their TMD number and topology. Class I holins typically have three TMDs, class II holins contain two TMDs, and class III holins possess only one TMD. Most phages encode class I or II holins, whereas class III holins, with a single TMD, are less common, as reported by Moussa et al. (2014). The N-terminal of PROKKA_03659 was found to be positioned in extracellular region at 22nd amino acid and C-terminal positioned in the cytoplasmic region of 41st amino acid. Moreover, PROKKA_04292 and PROKKA_04422 belong to class II holin proteins with two transmembrane domains. Here N-terminal positioned in extracellular region at 34^th^ amino acid and C-terminal positioned the cytoplasmic region of 51st amino acid of PROKKA_04292. However, in case of PROKKA_04422, the N-terminal position has been found in extracellular region at 37th amino acid and C-terminal was found in the cytoplasmic region of 54th amino acid (as shown in Figure 7). Based on their TMD composition, PROKKA_03659 likely belongs to class III, while PROKKA_04292 and PROKKA_04422 may align with class II holins.

## Discussion

The ASEC2201 coliphage, cataloged under accession number SRX17770782, in NCBI SRA represents a significant addition to our understanding of bacteriophage biology, particularly in the context of antibiotic resistance. Isolated from drug-resistant clinical strains of *E. coli* ASEC2201 underwent comprehensive characterization and whole-genome sequencing, with the resulting genomic data made available in the NCBI database. This foundational work allows for further exploration of the phage’s functional components, particularly its lytic enzymes, which play a crucial role in the phage’s ability to lyse bacterial cells. Among the lytic enzymes identified in ASEC2201, three holin proteins PROKKA_03659, PROKKA_04292, and PROKKA_04422were characterized. Holins are integral membrane proteins that facilitate the release of endolysins into the bacterial periplasm, ultimately leading to cell lysis. The presence of active promoters associated with these proteins indicates significant transcriptional activity, underscoring their importance in the bacteriophage-mediated lysis system. Specifically, the identified promoters at positions 112, 177, and 186 for each respective protein suggest a well-regulated expression system that enables the phage to effectively target and dismantle resistant bacterial strains. Stability indices were calculated for these holins, revealing that all three proteins exhibit stability, with values of 28.95 for PROKKA_03659, 5.53 for PROKKA_04292, and 2.76 for PROKKA_04422.

A higher stability index often correlates with the potential efficacy of proteins in therapeutic applications, as stable proteins are less likely to denature under physiological conditions (Heselpoth et al., 2015; Abdelghafar et al., 2023). This stability is crucial for their functionality, especially when considering the potential therapeutic applications of phage therapy against resistant bacterial infections (Zaki et al., 2023; Bagińska et al., 2024). The theoretical isoelectric points (pI) for these holins fall within the basic range, with values of 9.43, 9.52, and 9.57, respectively. This basicity suggests that the functional roles of these proteins may be influenced by pH conditions, which is particularly relevant in the variable microenvironments within the human body. The aliphatic indices of these holins, all exceeding 66, indicate a high degree of thermal stability, which enhances their potential as therapeutic agents. The specific values of 85.49 for PROKKA_03659, 97.65 for PROKKA_04292, and 92.11 for PROKKA_04422 reflect their structural resilience, making them promising candidates for further study. Wang et al. (2025) have reported that the holin protein from *Acinetobacter nosocomialis* phage XC1 (ORF: XC1_81) exhibited an aliphatic index of 109.12 and an instability index of 21.36, concluding high thermal stability and structural resilience of protein. Additionally, the GRAVY index values for these proteins, all greater than 0 (specifically, 0.183 for PROKKA_03659, 0.101 for PROKKA_04292, and 0.61 for PROKKA_04422), suggest that they are hydrophobic in nature. This hydrophobic characteristic indicates a likelihood of reduced water interaction and potential membrane association, which is crucial for their function in transmembrane lysis. The presence of a hydrophobic region in holin proteins is critical for their ability to interact with the inner bacterial membrane, ultimately leading to the formation of pores that allow endolysins to access the peptidoglycan layer. Through domain analysis, these holins were classified within the Phage_holin_2_1 superfamily (Reddy and Saier, 2013) characterized by small hydrophobic proteins that are essential for the lytic cycle of bacteriophages. The identification of this superfamily highlights the evolutionary conservation of these proteins and their critical roles in phage biology.

The use of DeepTMHMM in the transmembrane domain (TMD) analysis of holin proteins in bacteriophage ASEC2201 represents a significant advancement in accurately predicting membrane topology. Previous researches have utilized various methods to predict holin TMDs. For instance, studies on *E. coli* phage ECP26 employed TMHMM to identify holin candidates based on TMD presence and length, leading to the identification of ORF151 as a class II holin. Similarly, investigations into Lactobacillus phages revealed a prevalence of class III holins with single TMDs, highlighting the diversity in holin structures across different phage species (Park et al., 2022). The findings from ASEC2201 holin analysis align with structural predictions of other phage-derived therapeutic proteins. For example, the mycobacteriophage D29 holin has been shown to possess two TMDs with a coiled-coil C-terminal region, essential for efficient host cell lysis. Similarly, the phage φ21 holin S^21 utilizes its second TMD to form small membrane pores, facilitating lysis (Bavda et al., 2021).

Integrating cell-penetrating peptides (CPPs) with holins enhances phage mediated lysis against intracellular bacterial infections. Zhao et al. (2023) engineered Salmonella phages displaying CPPs, improving their uptake by epithelial cells and effectively eradicating intracellular bacteria without harming host cells. Similarly, Wang et al. (2018) fused a CPP to the phage lysin JDlys, facilitating its entry into keratinocytes and eliminating intracellular MRSA, thereby accelerating wound healing in mice. The three holins of ASEC2201 under study, possess specific N-terminal regions which exhibit CPP-like properties, suggesting potential for delivering holin activity directly to intracellular pathogens.

The structural characterization of the three identified holin proteins of ASEC2201 revealed that the holin sequences were aligned with high-confidence templates (e.g., A0A655MZF6.1. A, P77237.1. A, P0A9R2.1. A), yielding a 100% probability and an exceptionally low E-value of 2.4 × 10^−116^. This indicates a robust structural similarity to known holin proteins, reinforcing the hypothesis that these sequences encode functional holins. Subsequent validation of Ramachandran plot analysis revealed high percentages in the favored regions underscore the structural integrity of the holins, suggesting that they are well-folded and likely to be functionally active. The comprehensive structural analysis of the ASEC2201 holins provides a solid foundation for their potential application in phage therapy, particularly against multidrug-resistant *E.coli* strains. Their well-defined tertiary structures and validation through Ramachandran plot analysis affirm their suitability as candidates for drug delivery vectors (Hou et al., 2018; Bavda et al., 2021; Pollenz et al., 2022; Li et al., 2023; Tang et al., 2025).

## Conclusion

In summary, the detailed genomic and biophysical characterization of ASEC2201 holins (PROKKA_03659, PROKKA_04292, PROKKA_04422) underscores their utility as modular membrane–disruption devices in engineered antimicrobial platforms. Their confirmed transmembrane topologies, robust stability profiles, and CPP-like N-terminal signals position these proteins not only as precise lytic effectors in phage therapy against multidrug-resistant *E. coli*, but also as versatile carriers for intracellular cargo delivery. By leveraging strong, promoter-driven expression and the inherent specificity of holin–endolysin pairs, next-generation biotechnological applications could integrate holin modules into synthetic vesicles, fusion proteins, or conjugated nanocarriers, enabling targeted breaching of bacterial membranes or host-cell endosomal compartments. Ultimately, ASEC2201 holins represent a powerful toolkit for designing bespoke antimicrobial and drug-delivery systems that combine genetic programmability with membrane-permeabilizing precision.

## Data Availability

The original contributions presented in the study are publicly available. This data can be found at: https://www.ncbi.nlm.nih.gov/, accession number: SRX17770783.
