# Supplementary material for: Characterization of holins, the membrane proteins of coliphage ASEC2201: a genomewide in silico approach
Source: Front Microbiol. 2025 Jul 9;16:1550594. doi: 10.3389/fmicb.2025.1550594 (PMC12283622; doi:10.3389/fmicb.2025.1550594)
Supplement: Supplementary file 1 [file Supplementary_file_1.docx]

**Supplementary Tables and Figures**

**Suppl. Table 1.** FASTA sequences of PROKKA_03659, PROKKA_04292 and PROKKA_04422

| Holins | FASTA Sequences |
| --- | --- |
| PROKKA_03659 | >PROKKA_03659 holin  ATGAAAGATTTTTTAAACGCTGCAACTTCCGGCACTGGCGGAGCGTCAATCACTGGCGCGGTAACTGGTCAAACAACTATTGCAATAGCCAGCTTCGTTTTGATGGCTGCATTTGGTATGTGGGGCGCTTATCTTCGCTGGCGTGATAGTAAGGCGCTACGTGACGCGCTTGAATGCGGTGATATTAAGAAAGCTATTGAGATCAGAGGTAAATAA |
|  | >PROKKA_03659 holin  MKDFLNAATSGTGGASITGAVTGQTTIAIASFVLMAAFGMWGAYLRWRDSKALRDALECGDIKKAIEIRGK |
| PROKKA_04292 | >PROKKA_04292 holin  ATGGATAAGTTAACAACAGGTGTTGCCTATGGCACATCGGCGGGTAATGCTGGTTTCTGGGCATTGCAGTTACTCGATAAAGTAACTCCGTCACAGTGGGCTGCAATCGGTGTGCTGGGTAGCCTGGTTTTTGGCCTGCTGACGTATCTGACAAATCTTTATTTCAAGATTAAAGAAGACAGGCGTAAGGCTGCGAGAGGAGAGTAA |
|  | >PROKKA_04292 holin  MDKLTTGVAYGTSAGNAGFWALQLLDKVTPSQWAAIGVLGSLVFGLLTYLTNLYFKIKEDRRKAARGE |
| PROKKA_04422 | >PROKKA_04422 holin  ATGAAATCCATGGATAAGTTAACAACGGGTGTCGCCTATGGCACCTCAGCAGGTAGTGCCGGGTACTGGTTTTTACAGCTGCTAGATAAAGTCACTCCCTCACAGTGGGCAGCAATAGGTGTGCTGGGTAGCCTGGTATTTGGCCTGCTGACGTACCTGACAAACCTTTATTTCAAGATTAAAGAAGATAAGCGCAAGGCTGCGAGAGGTGAATAA |
|  | >PROKKA_04422 holin  MKSMDKLTTGVAYGTSAGSAGYWFLQLLDKVTPSQWAAIGVLGSLVFGLLTYLTNLYFKIKEDKRKAARGE |

**Suppl. Table 2.** Amino acid composition of ASEC2201 Holins

| **Amino Acid (%)** | **PROKKA_03659** | **PROKKA_4294** | **PROKKA_4421** |
| --- | --- | --- | --- |
| **Ala (A)** | 16.9 | 11.8 | 9.9 |
| **Arg (R)** | 5.6 | 4.4 | 2.8 |
| **Asn (N)** | 1.4 | 2.9 | 1.4 |
| **Asp (D)** | 5.6 | 4.4 | 4.2 |
| **Cys (C)** | 1.4 | 0.0 | 0.0 |
| **Gln (Q)** | 1.4 | 2.9 | 2.8 |
| **Glu (E)** | 2.8 | 2.9 | 2.8 |
| **Gly (G)** | 12.7 | 11.8 | 11.3 |
| **His (H)** | 0.0 | 0.0 | 0.0 |
| **Ile (I)** | 8.5 | 2.9 | 2.8 |
| **Leu (L)** | 7.0 | 14.7 | 14.1 |
| **Lys (K)** | 7.0 | 7.4 | 9.9 |
| **Met (M)** | 4.2 | 1.5 | 2.8 |
| **Phe (F)** | 4.2 | 4.4 | 4.2 |
| **Pro (P)** | 0.0 | 1.5 | 1.4 |
| **Ser (S)** | 5.6 | 4.4 | 7.0 |
| **Thr (T)** | 8.5 | 8.8 | 8.5 |
| **Trp (W)** | 2.8 | 2.9 | 2.8 |
| **Tyr (Y)** | 1.4 | 4.4 | 5.6 |
| **Val (V)** | 2.8 | 5.9 | 5.6 |
| **Pyl (O)** | 0.0 | 0.0 | 0.0 |
| **Sec (U)** | 0.0 | 0.0 | 0.0 |
| **(B)** | 0.0 | 0.0 | 0.0 |
| **(Z)** | 0.0 | 0.0 | 0.0 |
| **(X)** | 0.0 | 0.0 | 0.0 |

**Suppl. Table 3.** Secondary structure elements of ASEC2201 holins as predicted by SOPMA (Self-Optimized Prediction Method with Alignment)

| **Secondary Structure Elements** | **>PROKKA_03659** | **>PROKKA_04292** | **>PROKKA_04422** |
| --- | --- | --- | --- |
| **Alpha helix (Hh)** | 45 is 63.38% | 38 is 55.88% | 44 is 61.97% |
| **3_10_helix (Gg)** | 0 | 0 | 0 |
| **Pi helix (Ii)** | 0 | 0 | 0 |
| **Beta bridge (Bb)** | 0 | 0 | 0 |
| **Extended strand (Ee)** | 10 is 14.08% | 0 | 1 is 1.41% |
| **Beta turn (Tt)** | 0 | 0 | 0 |
| **Bend region (Ss)** | 0 | 0 | 0 |
| **Random coil (Cc)** | 16 is 22.54% | 30 is 44.12% | 26 is 36.62% |
| **Ambiguous states** | 0 | 0 | 0 |
| **Other states** | 0 | 0 | 0 |

**Suppl. Table 4.** Ramachandran plot statistics of the modeled holins of ASEC2201

| **Ramachandran Plot Statistics** | **>PROKKA_03659** | **>PROKKA_04292** | **>PROKKA_04422** |
| --- | --- | --- | --- |
| **Residues in the most favored regions (A, B, L)** | 36 (90%) | 31 (96.9%) | 31 (91.2%) |
| **Residues in additional allowed regions (a, b, l, p)** | 3 (7.5%) | 0 (0.0%) | 3 (8.8%) |
| **Residues in generously allowed regions (~a, ~b, ~l, ~p)** | 0 (0.0%) | 0 (0.0%) | 0(0.0%) |
| **Residues in disallowed regions** | 1 (2.5%) | 1 (3.1%) | 0 (0.0%) |
| **Number of non-glycine and non-proline residues** | 40 (100.0%) | 32 (100.0%) | 34 (100.0%) |
| **Number of end residues (excl. Gly and Pro)** | 2 | 2 | 2 |
| **Number of glycine residues (shown as triangles)** | 4 | 3 | 3 |
| **Number of proline residues** | 0 | 1 | 1 |
| **Total number of residues** | 46 | 38 | 40 |


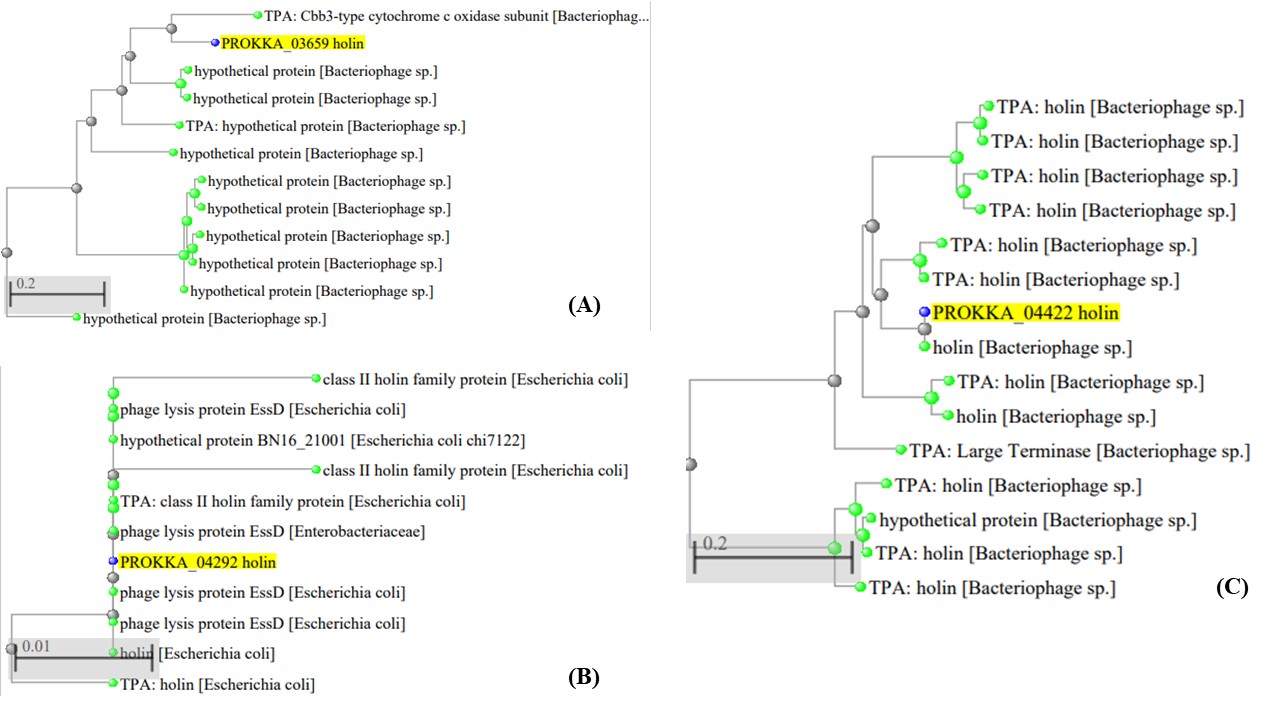


**Suppl. Figure 1.** Phylogenetic analysis of PROKKA_03659 (A), PROKKA_04292 (B) and PROKKA_04422 (C) by using neighbor joining method in MegaX

**
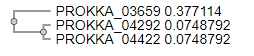
**

**Suppl. Figure 2. Phylogenetic tree building of PROKKA_03659, PROKKA_04292 and PROKKA_04422 by using Mega X**


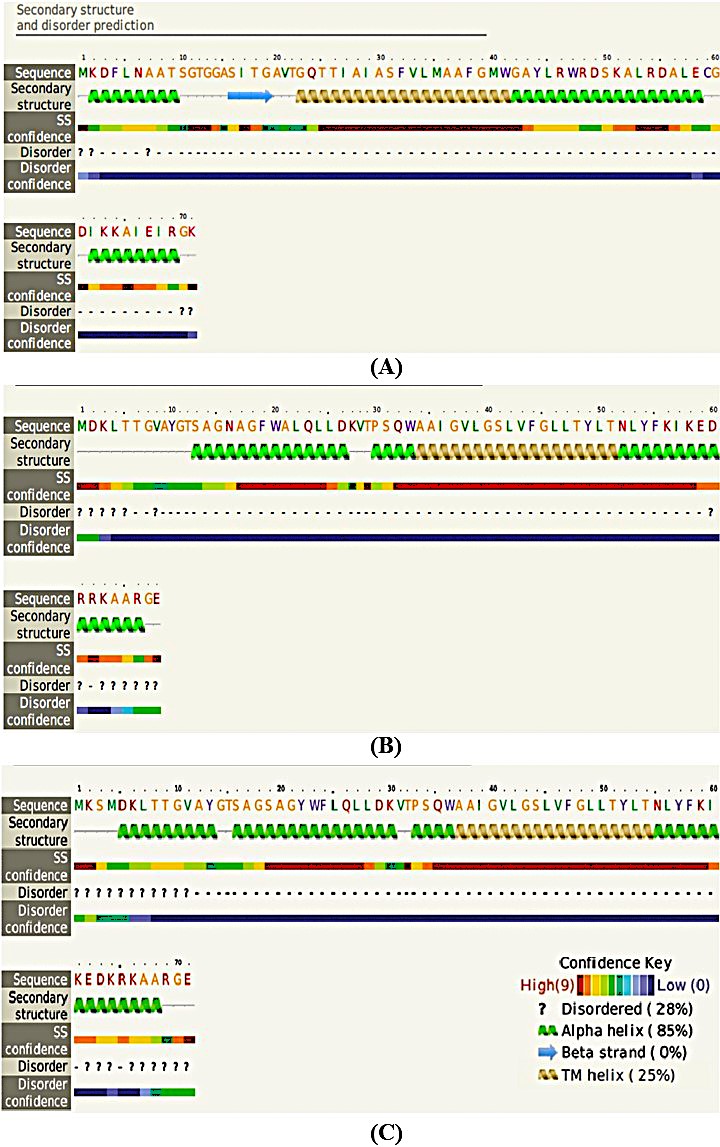


**Suppl. Figure 3.** Protein Homology/analogy Recognition by Phyre2 of PROKKA_03659 (A), PROKKA_04292 (B) and PROKKA_04422 (C)
